# Evaluation of the Ki-67 Labeling Index as a Prognostic Stratification Factor in Non-High-Risk Gastrointestinal Stromal Tumors

**DOI:** 10.7759/cureus.93440

**Published:** 2025-09-28

**Authors:** Midori Wakiya, Akira Okimura, Hiroshi Hirano, Satoru Tabuchi, Shigeyuki Kawachi, Hideaki Hirai, Yuichi Nagakawa, Tatsuhiko Miyazaki, Toshitaka Nagao, Munehide Nakatsugawa

**Affiliations:** 1 Department of Diagnostic Pathology, Tokyo Medical University Hachioji Medical Center, Hachioji, JPN; 2 Department of Digestive and Transplantation Surgery, Tokyo Medical University Hachioji Medical Center, Hachioji, JPN; 3 Department of Anatomic Pathology, Tokyo Medical University, Shinjuku, JPN; 4 Department of Gastrointestinal and Pediatric Surgery, Tokyo Medical University, Shinjuku, JPN; 5 Department of Pathology, Gifu University Hospital, Gifu, JPN

**Keywords:** gastrointestinal stromal tumor (gist), ki-67 labeling index, modified nih risk classification, non-high-risk, prognosis

## Abstract

Aim

This study aimed to evaluate the Ki-67 labeling index (LI) as a prognostic factor for recurrence in patients with non-high-risk gastrointestinal stromal tumors (GISTs), as defined by the modified NIH risk classification.

Patients and methods

In this retrospective study, 72 patients with GISTs who had undergone complete tumor resection and received no adjuvant therapy until recurrence were included. The Ki-67 LI and mitotic count were assessed in hotspot sections of each tumor.

Results

According to the modified NIH risk classification, 54 tumors were categorized as non-high-risk (very low-, low-, or intermediate-risk) and 18 as high-risk. During follow-up, recurrence occurred in seven (13.0%) non-high-risk cases and nine (50.0%) high-risk cases. Receiver operating characteristic analysis for recurrence identified an optimal Ki-67 LI cutoff of 3.0% for non-high-risk GISTs. Among non-high-risk cases, recurrence occurred in seven of 26 (26.9%) with Ki-67 LI ≥ 3.0%, whereas none of the 28 with Ki-67 LI < 3.0% experienced recurrence. Kaplan-Meier analysis of recurrence-free survival, stratified by this cutoff, showed significantly worse outcomes for the non-high-risk group with Ki-67 LI ≥ 3.0%.

Conclusions

These findings suggest that Ki-67 LI may serve as a useful stratification factor in patients with non-high-risk GISTs. In particular, applying Ki-67 LI stratification may help identify non-high-risk patients who require closer surveillance.

## Introduction

Gastrointestinal stromal tumor (GIST) is the most common mesenchymal neoplasm of the gastrointestinal tract, arising from the interstitial cells of Cajal or their precursors [1,2]. Although most GISTs occur in the stomach, they can also develop in the small intestine, colon, and extragastrointestinal sites such as the omentum and mesentery [1,3]. The standard treatment for localized GIST is complete surgical resection, which remains the only curative option [4]. However, the risk of recurrence after surgery is a significant clinical concern, with up to 50% of patients with high-risk features experiencing recurrence within five years [5].

To address this issue, adjuvant therapy with the tyrosine kinase inhibitor imatinib has been shown to significantly improve recurrence-free survival (RFS), particularly in high-risk patients [6]. Nevertheless, this therapy is associated with potential adverse effects and requires long-term administration, underscoring the importance of accurate patient stratification [4,7]. The most widely used tool for this purpose is the modified NIH risk classification, which categorizes patients according to tumor size, mitotic count, and tumor location [2,5,8,9]. While this system is the most effective available tool for identifying high-risk patients, recurrence can still occur in individuals classified as non-high risk [2,5,8,9]. This presents a clinical challenge: although most non-high-risk patients have favorable outcomes and can avoid adjuvant therapy, a small but important subset still develops recurrence, reflecting prognostic heterogeneity within this group [5]. Refining risk assessment for these patients is therefore critical to identify those who may benefit from closer monitoring or consideration of therapy.

Several additional prognostic indicators have been proposed to further stratify risk in non-high-risk patients, including SOCS6 [10], sarcopenia [11], miR-196a and HOTAIR [12], LINE-1 hypomethylation [13], and PPH [14]. Among these, the Ki-67 protein, a well-established marker of cellular proliferation, has received considerable attention. Numerous studies and meta-analyses have demonstrated that a high Ki-67 labeling index (LI) is an independent prognostic factor associated with worse recurrence-free and overall survival in GISTs [15-28]. However, most prior studies have focused on broad GIST cohorts or specifically on high-risk populations, and reported cutoff values for Ki-67 LI vary widely. The prognostic significance and optimal cutoff value of Ki-67 in the non-high-risk GIST population remain less clearly defined.

The present study, therefore, aimed to evaluate the potential of Ki-67 as a stratification factor specifically in non-high-risk GISTs, as defined by the modified NIH risk classification.

## Materials and methods

Patient information

In this retrospective study, a total of 72 patients with GIST treated at Hachioji Medical Center, Tokyo Medical University, and affiliated hospitals between 2006 and 2020 were enrolled. Inclusion required fulfillment of all the following criteria: a pathologically confirmed diagnosis of GIST, complete tumor resection, no adjuvant therapy prior to recurrence, and availability of complete clinicopathological and follow-up data. Tumors were stratified according to the modified NIH risk classification proposed by Joensuu [2]. The study was approved by the Institutional Review Board of Tokyo Medical University (approval T2024-0067). All patients provided consent for the use of tumor tissues and clinical data.

Ki-67 immunohistochemistry

**Figure 1:**
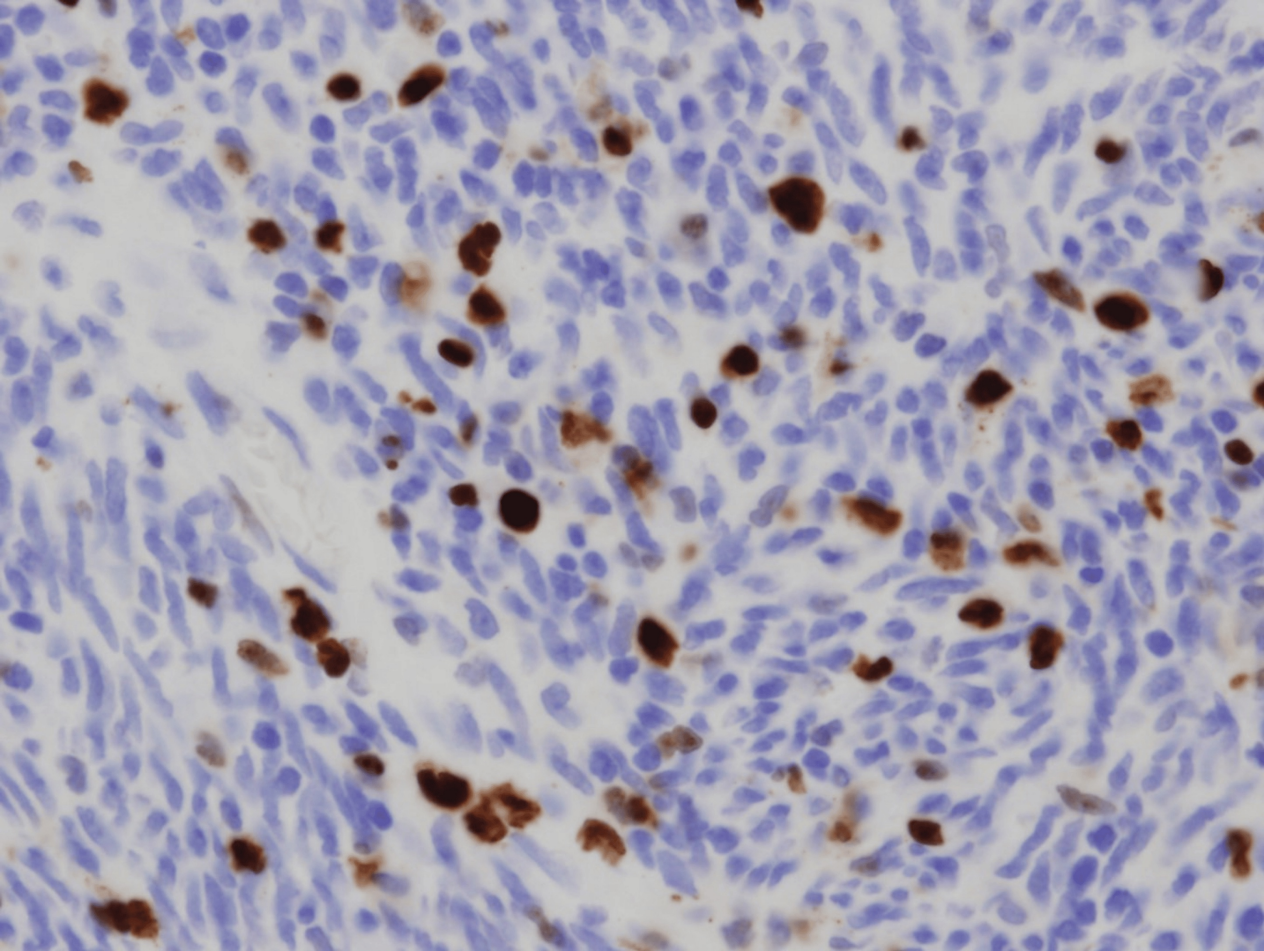
Representative image of Ki-67 immunohistochemical staining Brown-stained nuclei indicate Ki-67-positive tumor cells (original magnification, ×400).

Each tumor was sectioned at its maximal surface and fixed in 10% phosphate-buffered formalin (pH 7.4) immediately after surgical resection. After fixation for 12-24 hours, blocks containing the maximal cut surface were prepared, and each was cut into several 4-μm-thick sections. One section was stained with H&E, and another was used for immunohistochemical detection of Ki-67. Immunohistochemistry was performed using an automated system (BOND-III, Leica Biosystems, Nussloch, Germany) following heat-induced epitope retrieval (Epitope Retrieval Solution 2, 20 minutes). The primary anti-human Ki-67 mouse monoclonal antibody (clone MIB-1, Dako, Glostrup, Denmark) was applied at a 1:200 dilution (Figure 1).

Image analysis

Pathologic evaluation was performed by two independent pathologists. Entire slides were initially scanned at low magnification to identify areas of highest proliferative activity (“hotspots”). For mitotic counting, the number of mitoses was recorded in 50 consecutive high-power fields within the identified mitotic hotspot on H&E-stained slides. For Ki-67 assessment, an image of the representative Ki-67 hotspot was captured using a digital microscope camera (DP70; Olympus, Tokyo, Japan) at ×200 magnification. The Ki-67 LI was then quantified as the percentage of positive tumor cells among at least 500 tumor cells using a software package (e-count; Fujisawa, Tokyo, Japan). To ensure reproducibility and minimize inter-observer variability, any significant discrepancies in hotspot selection or final counts between the two pathologists were resolved by joint review and consensus.

Statistical analysis

Statistical analyses were performed using BellCurve for Excel, version 3.22 (SSRI, Tokyo, Japan). The cutoff value of the Ki-67 LI for discriminating between patients with and without recurrence in non-high-risk GISTs, as classified by the modified NIH risk classification, was determined using receiver operating characteristic (ROC) analysis. Recurrence-free survival (RFS) was defined as the interval between the date of surgery and the date of recurrence or the last follow-up examination. RFS was analyzed using the Kaplan-Meier method and compared with the log-rank test, with p < 0.05 considered statistically significant.

## Results

Characteristics of patients and tumors

When the 72 GISTs were classified according to the modified NIH risk classification, 54 were categorized as non-high risk and 18 as high risk. Within the non-high-risk group, 14 were classified as very low risk, 27 as low risk, and 13 as intermediate risk (Table 1).

**Table 1:** Stratification of GIST cases based on the modified NIH risk classification, including the number of GISTs and recurrences in each risk group No tumor rupture occurred in any of the cases. GIST, gastrointestinal stromal tumor; HPF, high-power field

Risk category	Primary tumor site	Tumor size (cm)	Mitotic index (/50 HPF)	GISTs (no.)	Recurrence (no.)
Very low	Any	≤2	≤5	14	1
Low	Any	2.1-5	≤5	27	2
Intermediate	Gastric	2.1-5	>5	2	1
	Any	<5	6-10	1	0
	Gastric	5.1-10	≤5	10	3
High	Tumor rupture	Any	Any	0	0
	Any	>10	Any	8	4
	Any	Any	>10	3	1
	Any	>5	>5	2	0
	Non-gastric	2.1-5	>5	0	0
	Non-gastric	5.1-10	≤5	5	4

In the non-high-risk group, 41 cases originated in the stomach and 13 in non-gastric sites. The male-to-female ratio was 32:22. The mean tumor size was 3.40 cm (median, 2.8 cm; range, 0.4-10.0 cm). The mean patient age was 66.2 years (median, 67 years; range, 40-87 years). The mean Ki-67 LI in the non-high-risk group was 5.51% (median, 2.61%; range, 0-50.5%).

In the high-risk group, 10 cases originated in the stomach and eight in non-gastric sites. The male-to-female ratio was 13:5. The mean tumor size was 8.98 cm (median, 8.0 cm; range, 2.2-14.0 cm). The mean Ki-67 LI in this group was 8.27% (median, 4.87%; range, 0.17-40.5%).

Ki-67 LI as a stratifying factor in the non-high-risk group

**Figure 2:**
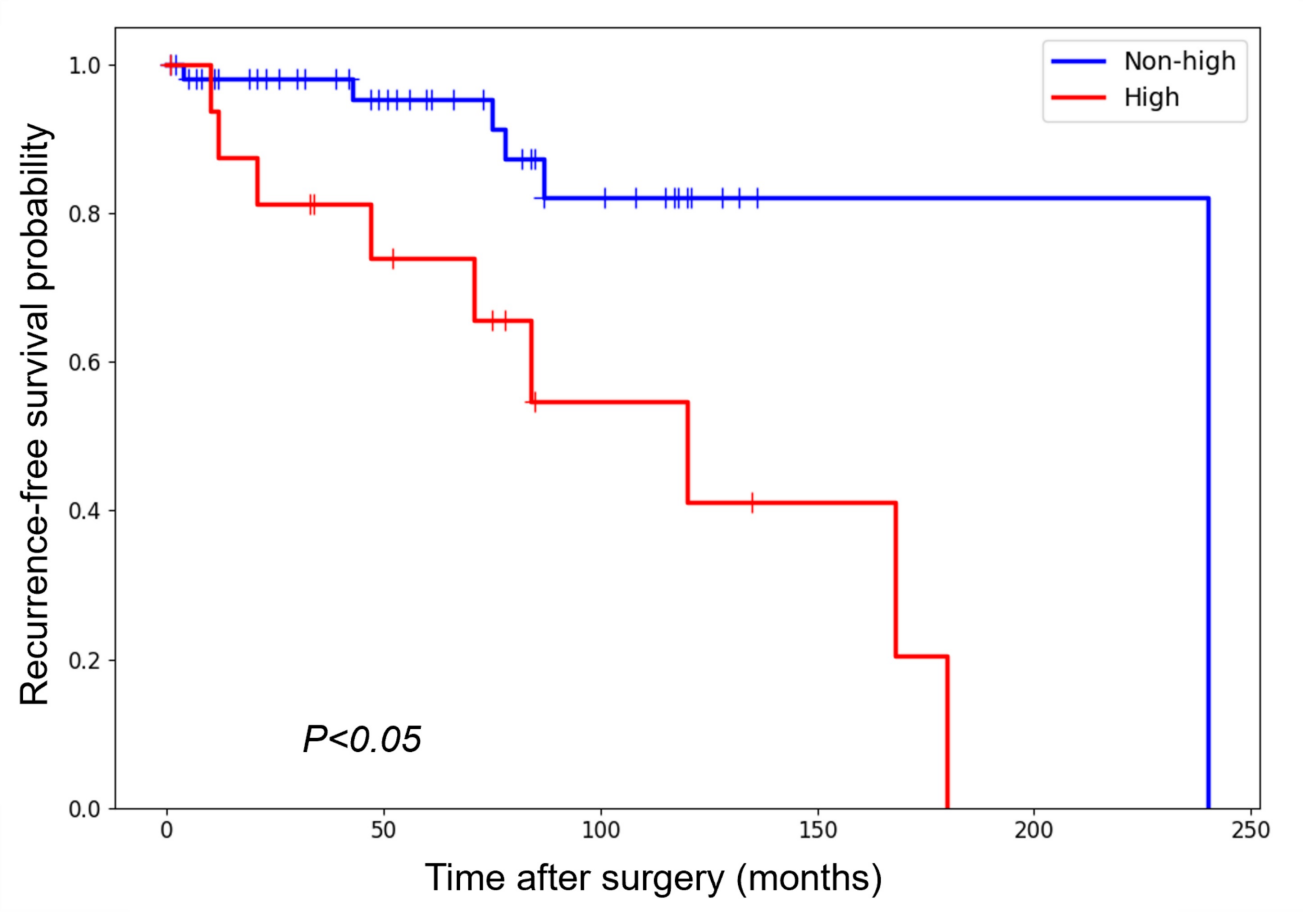
Kaplan-Meier curves of RFS (%) for patients with high-risk and non-high-risk GISTs, classified according to the modified NIH risk classification GIST, gastrointestinal stromal tumor; RFS, recurrence-free survival

Kaplan-Meier curve analysis of RFS showed a significantly worse prognosis for patients with high-risk GISTs compared with those with non-high-risk GISTs, including very low-, low-, and intermediate-risk groups (Figure 2).

**Figure 3:**
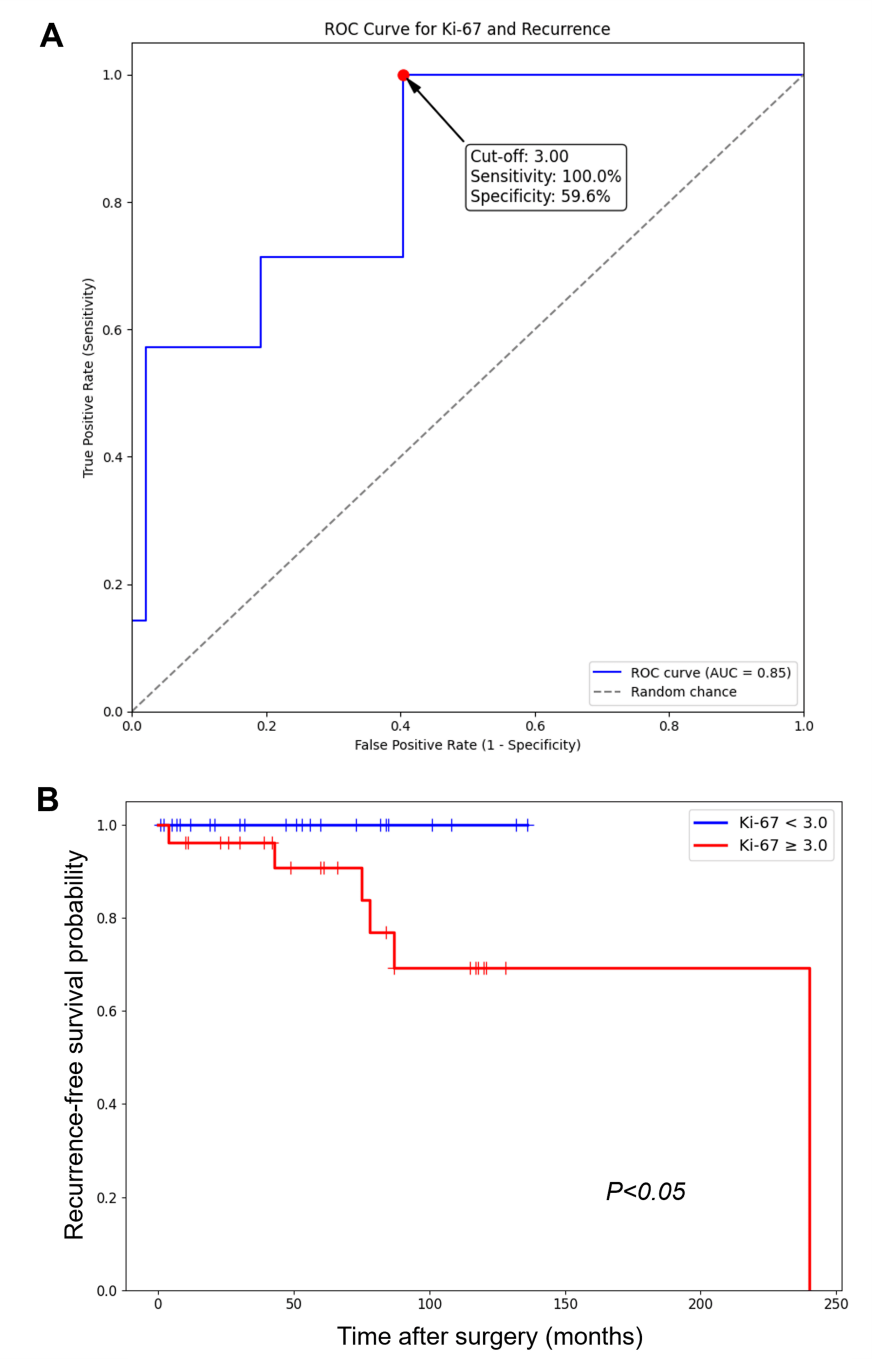
Prognostic value of the Ki-67 LI in non-high-risk GISTs (A) ROC analysis for recurrence in patients with non-high-risk GISTs. (B) Kaplan-Meier curves of RFS (%) for non-high-risk patients, stratified by the cutoff value (3.0%). GIST, gastrointestinal stromal tumor; LI, labeling index; RFS, recurrence-free survival; ROC, receiver operating characteristic

However, recurrence occurred in seven of 54 patients (13.0%) in the non-high-risk group. Using ROC analysis, the optimal cutoff value for Ki-67 LI in this group was determined to be 3.0% (AUC = 0.85; Figure 3A), with a sensitivity of 100% and a specificity of 59.6%. Based on this cutoff, the 54 non-high-risk patients were stratified into two subgroups. In the high Ki-67 group (LI ≥ 3.0%), recurrence occurred in seven of 26 patients (26.9%). In contrast, in the low Ki-67 group (LI < 3.0%), no recurrence was observed among the 28 patients (0%). Kaplan-Meier analysis confirmed that RFS was significantly lower in patients with Ki-67 LI ≥ 3.0% compared with those with Ki-67 LI < 3.0% (Figure 3B).

## Discussion

Many studies have demonstrated that the Ki-67 LI is an independent prognostic factor for GISTs, although reported cutoff values vary widely, ranging from 1% to 22% [9-20]. Two studies have further suggested that Ki-67 LI can serve as an independent stratifying factor for patients with high-risk GISTs (per the modified NIH risk classification), particularly in identifying those at very high risk [15,20]. Combining Ki-67 LI with the modified NIH risk classification has been shown to improve patient selection for adjuvant chemotherapy compared with using the modified NIH criteria alone [15,20]. In those studies, the cutoff values were set at 6% or 8%. In the present study, however, we did not assess Ki-67 LI as a stratifying factor in high-risk GISTs because of the limited number of cases.

Liu et al. reported that a Ki-67 LI cutoff of 6% could stratify 603 non-high-risk GISTs (classified by the modified NIH criteria) in terms of overall survival, although the difference did not reach statistical significance [20]. Their cutoff was determined by ROC analysis for overall survival across both non-high-risk and high-risk groups. By contrast, our study specifically focused on non-high-risk patients and determined the cutoff value for recurrence. ROC analysis identified 3.0% as the optimal threshold, and Kaplan-Meier curve analysis confirmed the prognostic significance of this cutoff in RFS. The discrepancy between Liu et al. and our findings may reflect differences in the methodology used to determine cutoff values. Nonetheless, because our sample size was much smaller than that of Liu et al., validation in larger cohorts is essential to confirm the robustness of our results.

Limitations

This study has several limitations. First, as a retrospective analysis, it is inherently subject to selection bias and confounding factors that may not be fully controlled, unlike prospective studies. Second, the relatively small sample size may limit statistical power, reduce the precision of estimates, and affect the reliability of the findings. The identified cutoff of 3.0%, although statistically significant in our cohort, requires validation in larger, independent, multi-center studies before it can be adopted clinically. Finally, this study focused only on RFS; longer follow-up will be necessary to clarify the effect of Ki-67 LI on overall survival.

## Conclusions

This study demonstrates that the Ki-67 LI is a valuable prognostic biomarker for stratifying recurrence risk within the non-high-risk GIST population, as defined by the modified NIH criteria. A cutoff value of 3.0% effectively distinguished a subgroup of patients at significantly higher risk of recurrence. Specifically, no recurrences occurred among patients with a Ki-67 LI < 3.0%, whereas 26.9% of those with a Ki-67 LI ≥ 3.0% experienced recurrence. These findings suggest that Ki-67 LI may help identify higher-risk patients within the non-high-risk category, but confirmation in larger, prospective studies is required before clinical application.

## References

[1] Miettinen M, Lasota J (2011). Histopathology of gastrointestinal stromal tumor. J Surg Oncol.

[2] Joensuu H (2008). Risk stratification of patients diagnosed with gastrointestinal stromal tumor. Hum Pathol.

[3] Miettinen M, Lasota J (2006). Gastrointestinal stromal tumors: pathology and prognosis at different sites. Semin Diagn Pathol.

[4] Demetri GD, von Mehren M, Antonescu CR (2010). NCCN Task Force report: update on the management of patients with gastrointestinal stromal tumors. J Natl Compr Canc Netw.

[5] Joensuu H, Vehtari A, Riihimäki J (2012). Risk of recurrence of gastrointestinal stromal tumour after surgery: an analysis of pooled population-based cohorts. Lancet Oncol.

[6] Dematteo RP, Ballman KV, Antonescu CR (2009). Adjuvant imatinib mesylate after resection of localised, primary gastrointestinal stromal tumour: a randomised, double-blind, placebo-controlled trial. Lancet.

[7] Casali PG, Abecassis N, Aro HT (2018). Gastrointestinal stromal tumours: ESMO-EURACAN Clinical Practice Guidelines for diagnosis, treatment and follow-up. Ann Oncol.

[8] Joensuu H (2013). Gastrointestinal stromal tumors: risk assessment and adjuvant therapy. Hematol Oncol Clin North Am.

[9] Jang SH, Kwon JE, Kim JH (2014). Prediction of tumor recurrence in patients with non-gastric gastrointestinal stromal tumors following resection according to the modified National Institutes of Health criteria. Intest Res.

[10] Ouyang J, An T, Wang Y (2021). Down-regulation of SOCS6: an unfavorable prognostic factor for gastrointestinal stromal tumor proven by survival analysis. Diagn Pathol.

[11] Song H, Xiao X, Liu G, Zhou J (2022). Sarcopenia as a novel prognostic factor in the patients of primary localized gastrointestinal stromal tumor. BMC Cancer.

[12] Niinuma T, Suzuki H, Nojima M (2012). Upregulation of miR-196a and HOTAIR drive malignant character in gastrointestinal stromal tumors. Cancer Res.

[13] Igarashi S, Suzuki H, Niinuma T (2010). A novel correlation between LINE-1 hypomethylation and the malignancy of gastrointestinal stromal tumors. Clin Cancer Res.

[14] Uguen A, Conq G, Doucet L, Talagas M, Costa S, De Braekeleer M, Marcorelles P (2015). Immunostaining of phospho-histone H3 and Ki-67 improves reproducibility of recurrence risk assessment of gastrointestinal stromal tumors. Virchows Arch.

[15] Carrillo R, Candia A, Rodriguez-Peralto JL (1997). Prognostic significance of DNA ploidy and proliferative index (MIB-1 index) in gastrointestinal stromal tumors. Hum Pathol.

[16] Toquet C, Le Néel JC, Guillou L (2002). Elevated (≥10%) MIB-1 proliferative index correlates with poor outcome in gastric stromal tumor patients: a study of 35 cases. Dig Dis Sci.

[17] Wang X, Mori I, Tang W (2002). Helpful parameter for malignant potential of gastrointestinal stromal tumors (GIST). Jpn J Clin Oncol.

[18] Nakamura N, Yamamoto H, Yao T (2005). Prognostic significance of expressions of cell-cycle regulatory proteins in gastrointestinal stromal tumor and the relevance of the risk grade. Hum Pathol.

[19] Jiang J, Jin MS, Suo J, Wang YP, He L, Cao XY (2012). Evaluation of malignancy using Ki-67, p53, EGFR and COX-2 expressions in gastrointestinal stromal tumors. World J Gastroenterol.

[20] Lu C, Liu L, Wu X, Xu W (2013). CD133 and Ki-67 expression is associated with gastrointestinal stromal tumor prognosis. Oncol Lett.

[21] Zhao WY, Xu J, Wang M (2014). Prognostic value of Ki67 index in gastrointestinal stromal tumors. Int J Clin Exp Pathol.

[22] Wang H, Chen P, Liu XX (2014). Prognostic impact of gastrointestinal bleeding and expression of PTEN and Ki-67 on primary gastrointestinal stromal tumors. World J Surg Oncol.

[23] Basilio-de-Oliveira RP, Pannain VL (2015). Prognostic angiogenic markers (endoglin, VEGF, CD31) and tumor cell proliferation (Ki67) for gastrointestinal stromal tumors. World J Gastroenterol.

[24] Pyo JS, Kang G, Sohn JH (2016). Ki-67 labeling index can be used as a prognostic marker in gastrointestinal stromal tumor: a systematic review and meta-analysis. Int J Biol Markers.

[25] Sugita S, Hirano H, Hatanaka Y (2018). Image analysis is an excellent tool for quantifying Ki-67 to predict the prognosis of gastrointestinal stromal tumor patients. Pathol Int.

[26] Liu X, Qiu H, Zhang P (2018). Ki-67 labeling index may be a promising indicator to identify "very high-risk" gastrointestinal stromal tumor: a multicenter retrospective study of 1022 patients. Hum Pathol.

[27] Li J, Wang AR, Chen XD, Pan H, Li SQ (2022). Ki67 for evaluating the prognosis of gastrointestinal stromal tumors: a systematic review and meta-analysis. Oncol Lett.

[28] Wang JP, Liu L, Li ZA, Wang Q, Wang XY, Lin J (2021). Ki-67 labelling index is related to the risk classification and prognosis of gastrointestinal stromal tumours: a retrospective study. Gastroenterol Hepatol.

